# Regional and Extraregional Pressure Pain Threshold Patterns in Tension-Type Headache and Low Back Pain: A Cross-Sectional Comparative Study

**DOI:** 10.3390/jcm15145368

**Published:** 2026-07-09

**Authors:** Aleksandar Kopitovic, Filip Katanic, Sandro Kalember, Nina Vico Katanic, Teodora Katanic, Aleksandra Lucic, Ivica Lalic, Svetlana Simic

**Affiliations:** 1 Faculty of Medicine, University of Novi Sad, 3 Hajduk Veljkova Street, 21000 Novi Sad, Serbia; aleksandar.kopitovic@mf.uns.ac.rs (A.K.); sandrokalember@yahoo.com (S.K.); nina.vico@mf.uns.ac.rs (N.V.K.); katanicteaa@gmail.com (T.K.); aleksandra.lucic-prokin@mf.uns.ac.rs (A.L.); svetlana.simic@mf.uns.ac.rs (S.S.); 2 Department of Neurology, University Clinical Center of Vojvodina, 1-9 Hajduk Veljkova Street, 21000 Novi Sad, Serbia; 3 Special Gynecology Hospital Ferona, 19 Sarplaninska Street, 21000 Novi Sad, Serbia; 4 Department of Anesthesia, Intensive Care and Pain Therapy, University Clinical Center of Vojvodina, 1-9 Hajduk Veljkova Street, 21000 Novi Sad, Serbia; 5 Health Center Novi Sad, 75 Bulevar Cara Lazara Street, 21000 Novi Sad, Serbia; 6 Faculty of Pharmacy, University Business Academy, 5 Trg Mladenaca Street, 21000 Novi Sad, Serbia; ivica.lalic@ffns.ac.rs

**Keywords:** tension-type headache, low back pain, pressure pain threshold, algometry, mechanical pain sensitivity

## Abstract

**Background/Objectives:** Tension-type headache (TTH) and low back pain (LBP) are common chronic pain disorders associated with altered mechanical pain sensitivity, but direct comparison of regional pressure pain threshold (PPT) patterns remains limited. **Methods:** This cross-sectional comparative study included 600 participants: 200 patients with TTH, 200 patients with LBP, and 200 healthy controls. PPT was assessed using digital pressure algometry in cranial, lumbar, and lower-extremity regions. Each point was tested three times, and mean regional values were calculated. Between-group differences were analyzed using one-way ANOVA. ANCOVA models were additionally adjusted for age, sex, and BMI, and patient-only multivariable regression models were adjusted for age, sex, BMI, pain intensity, and symptom duration. **Results:** Significant differences were observed in all examined regions (all *p* < 0.001). Patients with TTH had the lowest cranial PPT values, whereas patients with LBP showed the lowest lumbar PPT values. Both pain groups also demonstrated significantly reduced PPT values in anatomically distant regions compared with healthy controls. These regional differences remained significant after adjustment for relevant demographic, anthropometric, and clinical variables. **Conclusions:** TTH and LBP demonstrate distinct regional PPT profiles while sharing a pattern of extraregional mechanical hypersensitivity. These findings may be compatible with broader alterations in nociceptive processing, although central sensitization was not directly assessed.

## 1. Introduction

Tension-type headache (TTH) and low back pain (LBP) are highly prevalent pain disorders and major contributors to reduced quality of life, functional impairment, and healthcare burden. TTH is the most common primary headache disorder worldwide, with an estimated global prevalence of approximately 26%, while chronic TTH represents a clinically important subgroup because of its association with frequent pain, pericranial tenderness, and impaired daily functioning. LBP is the leading single cause of disability worldwide; approximately 619 million people were affected globally in 2020, and this number is projected to increase substantially in the coming decades [1,2,3,4,5,6,7,8].

Although TTH and LBP differ in anatomical localization, clinical presentation, and dominant peripheral nociceptive input, both conditions may be associated with altered mechanical pain sensitivity. In TTH, increased pericranial tenderness is one of the most reproducible clinical findings and may reflect enhanced sensitivity within cranio-cervical nociceptive pathways. In LBP, local mechanical hypersensitivity is commonly observed in lumbar paraspinal and related musculoskeletal structures. In both disorders, however, reduced pressure pain thresholds have also been reported at sites distant from the primary symptomatic region, suggesting that mechanical pain sensitivity may not be restricted to the clinically dominant area [5,9,10,11,12].

Pressure pain threshold (PPT) assessment using digital pressure algometry provides a standardized quantitative method for evaluating mechanical pain sensitivity. By applying gradually increasing pressure until the first perception of pain, PPT testing allows regional and extraregional sensitivity patterns to be compared across anatomical sites and clinical populations. Lower PPT values indicate greater mechanical pain sensitivity and may contribute to clinical phenotyping of chronic pain disorders [13,14,15,16,17].

Previous studies have examined PPT alterations in TTH and LBP separately, but direct comparisons between these two clinically distinct pain syndromes using the same algometry protocol remain limited. Such a comparison is clinically relevant because it may help distinguish syndrome-specific regional hypersensitivity from broader extraregional alterations in mechanical pain sensitivity. This distinction may clarify whether PPT reductions predominantly follow the primary symptomatic region or reflect a more widespread sensitivity pattern across distant body areas [13,14,17,18].

The aim of this study was to compare PPT values in cranial, lumbar, and lower-extremity regions among patients with TTH, patients with LBP, and healthy controls to determine patterns of regional and extraregional mechanical pain sensitivity. A secondary objective was to assess whether these clinically distinct pain syndromes exhibit predominantly local reductions in PPT or broader alterations in mechanical sensitivity that extend beyond the primary painful region.

## 2. Materials and Methods

This observational cross-sectional comparative study included 600 adult participants divided into three equal groups: 200 patients with TTH, 200 patients with LBP, and 200 healthy controls. Participants were recruited consecutively during routine outpatient evaluation.

The sample size was determined pragmatically based on the availability of consecutive eligible participants and the aim of obtaining equal group allocation across the three study groups. No formal a priori sample size calculation was performed.

The TTH group included patients with chronic tension-type headache diagnosed according to the International Classification of Headache Disorders, 3rd edition (ICHD-3), fulfilling criteria for headache occurring on ≥15 days per month for >3 months, with bilateral pressing or tightening pain of mild to moderate intensity, without aggravation by routine physical activity and without secondary headache features. Increased pericranial tenderness was clinically documented as an accompanying phenotypic characteristic. In ICD-11 classification, this entity corresponds to chronic tension-type headache [17,19].

The LBP group included patients with chronic low back pain defined as pain localized below the costal margin and above the inferior gluteal folds, persisting for longer than three months, without acute radicular syndrome, major neurological deficit, inflammatory spinal disease, or suspected secondary structural pathology. According to the ICD-11 and IASP classifications, the included patients predominantly corresponded to chronic primary low back pain [17,20,21].

Healthy controls were recruited among individuals without chronic pain complaints, without a history of chronic pain disorders, and without current musculoskeletal or neurological symptoms.

Exclusion criteria for all groups included age below 18 years, pregnancy, systemic inflammatory disease, malignant disease, major neurological disorders, generalized pain syndromes including fibromyalgia, previous major surgery involving the examined anatomical regions, and use of analgesic medication within 24 h before examination.

PPT was assessed using a handheld digital pressure algometer (DC 00408 AlgoMed, Medoc Ltd., Ramat Yishai, Israel; compliant with 93/42/EEC MDD) with a 1 cm^2^ probe. No separate data-acquisition software with an independent version number was used; PPT values were automatically recorded by the device at the moment of response-button activation. The probe was positioned perpendicular to the skin surface, and pressure was gradually increased at a constant rate of approximately 1 kg/s. Participants were instructed to press the response button at the first moment when the sensation of pressure became painful. At the moment of button press, pressure application was stopped, and the PPT value was automatically recorded by the device. The resulting value was expressed in kilopascals (kPa). Lower PPT values indicated greater mechanical pain sensitivity. Measurements were performed according to commonly used quantitative sensory testing protocols. All PPT measurements were performed by a trained examiner familiar with digital pressure algometry and the standardized anatomical testing protocol. The examiner was not blinded to group allocation [22,23,24,25,26,27].

PPT assessment was performed in three anatomical regions using predefined bilateral anatomical points derived from previously published PPT protocols for chronic headache and LBP studies. Measurements were performed according to a standardized regional order, beginning with cranial sites, followed by lumbar sites, and then lower-extremity sites. Cranial assessment points included the temporalis, masseter, frontalis, and upper trapezius muscles and were selected according to previously published cranio-cervical PPT protocols used in TTH studies. Lumbar assessment included bilateral paraspinal points at L1–L5. Lower-extremity assessment included predefined bilateral points along the lower leg to evaluate anatomically distant pressure-pain sensitivity [22,23,24,28,29].

Each anatomical point was tested three consecutive times, with approximately 60 s between repeated measurements at the same site to minimize temporal summation, local sensitization, and local tissue effects. For each anatomical point, the arithmetic mean of the three obtained values was calculated. For bilateral anatomical sites, left- and right-sided values were averaged. Regional PPT values were subsequently expressed as the mean value of all examined points within the corresponding anatomical region [22,23,25].

Age, sex, body mass index, pain intensity, symptom duration, and headache frequency (in the TTH group) were additionally recorded for secondary analyses.

Pain intensity was recorded as a patient-reported clinical variable using an 11-point Numerical Rating Scale (NRS), ranging from 0 (no pain) to 10 (worst imaginable pain). The NRS was used to characterize the clinical status of the patient groups. In contrast, the primary outcome of the study was the pressure pain threshold (PPT), expressed in kilopascals (kPa), measured using digital pressure algometry at local painful and anatomically distant reference points. Patients were asked to rate the intensity of their primary pain condition. In healthy controls, pain intensity was not assessed because these participants had no history of or evidence of a painful condition on clinical evaluation. Functional disability scales were not included in the correlation analyses in the present study, as the primary objective was to compare regional and extraregional mechanical pain sensitivity rather than functional impairment [30].

Normality of distribution was assessed using descriptive distribution parameters and visual inspection of distribution plots. Continuous variables were reported as mean ± standard deviation. Between-group comparisons of unadjusted PPT values were performed using one-way analysis of variance (ANOVA), followed by Tukey’s post hoc test.

To account for baseline differences in demographic and anthropometric variables, analysis of covariance (ANCOVA) models was performed for each regional PPT outcome, with study group as the fixed factor and age, sex, and body mass index as covariates. Adjusted means, 95% confidence intervals, adjusted *p* values, and partial eta-squared values were reported.

Because pain intensity and symptom duration were not applicable to healthy controls, additional patient-only multivariable regression models were performed in the TTH and LBP groups. These models included diagnosis as the main independent variable and were adjusted for age, sex, BMI, pain intensity, and symptom duration.

Associations between clinical variables and PPT values were explored using correlation analysis. A *p*-value below 0.05 was considered statistically significant. Statistical analyses were performed using IBM SPSS Statistics, version 26.0 (IBM Corp., Armonk, NY, USA).

The study was conducted in accordance with the principles of the Declaration of Helsinki and approved by the Ethics Committee of the Clinical Center of Vojvodina (protocol No. 00-81/1122; approved on 30 December 2016). Written informed consent was obtained from all participants prior to inclusion in the study.

## 3. Results

A total of 600 participants were included in the analysis, with 200 subjects in each group (TTH, LBP, and healthy controls). Baseline demographic and clinical characteristics, together with regional PPT values, are presented in Table 1.

PPT values in all examined regions showed approximately normal distribution, supporting the use of parametric statistical methods.

### 3.1. Primary Outcomes

#### 3.1.1. Regional Specificity of Pressure Pain Thresholds

**Figure 1:**
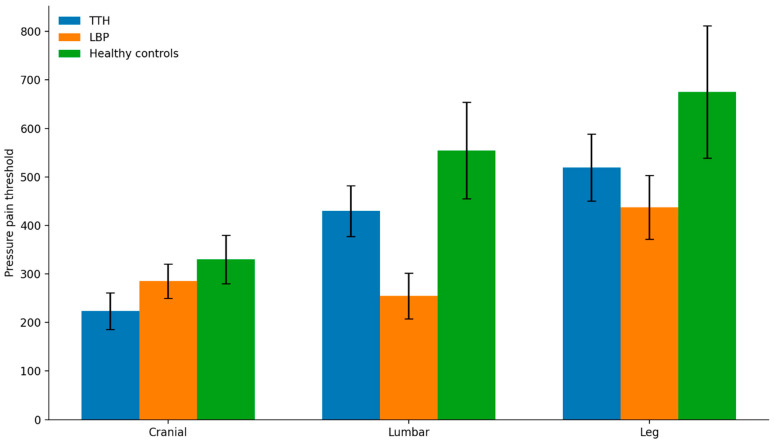
Mean regional pressure pain threshold values expressed in kilopascals (kPa) in cranial, lumbar, and lower-extremity regions across study groups. Error bars represent standard deviation.

Significant region-specific differences in PPT were observed between groups (Table 1 and Table 2; Figure 1).

In the cranial region, the lowest values were recorded in the TTH group (223.4 ± 37.6), followed by the LBP group (285.0 ± 35.0), while healthy controls had the highest thresholds (329.8 ± 50.2) (*p* < 0.001).

In the lumbar region, the lowest thresholds were observed in the LBP group (254.5 ± 47.0), compared with the TTH group (429.6 ± 52.3) and healthy controls (554.4 ± 99.6) (*p* < 0.001).

In the lower extremity region, PPT remained lowest in the LBP group (437.4 ± 65.6), intermediate in the TTH group (519.3 ± 69.3), and highest in healthy controls (675.3 ± 136.5) (*p* < 0.001).

#### 3.1.2. Adjusted Analyses of Regional PPT Values

In ANCOVA models adjusted for age, sex, and BMI, the study group remained significantly associated with PPT values in all examined regions. The adjusted group effect was significant for cranial PPT (F = 305.10, *p* < 0.001, partial η^2^ = 0.507), lumbar PPT (F = 533.90, *p* < 0.001, partial η^2^ = 0.643), and lower-extremity PPT (F = 195.45, *p* < 0.001, partial η^2^ = 0.397). Adjusted mean PPT values confirmed the same regional pattern observed in the unadjusted analysis: patients with TTH had the lowest cranial PPT values, patients with LBP had the lowest lumbar PPT values, and healthy controls had the highest PPT values across all regions (Table 3).

In additional patient-only multivariable regression models including the TTH and LBP groups, diagnosis remained significantly associated with regional PPT values after adjustment for age, sex, BMI, pain intensity, and symptom duration. Compared with TTH, LBP was associated with higher cranial PPT values (B = 63.9 kPa, 95% CI 55.0 to 72.8, *p* < 0.001), lower lumbar PPT values (B = −173.7 kPa, 95% CI −185.8 to −161.5, *p* < 0.001), and lower PPT values in the lower-extremity region (B = −81.4 kPa, 95% CI −97.9 to −64.9, *p* < 0.001) (Table 4).

#### 3.1.3. Reduced Thresholds in Distant Regions

Both pain groups also demonstrated significantly reduced PPT values in anatomically distant regions compared with healthy controls (Figure 1).

Patients with TTH showed lower lumbar and lower-extremity thresholds, while patients with LBP also demonstrated lower cranial thresholds than controls (all *p*-values < 0.001).

#### 3.1.4. Lowest Values in the Clinically Affected Region

**Figure 2:**
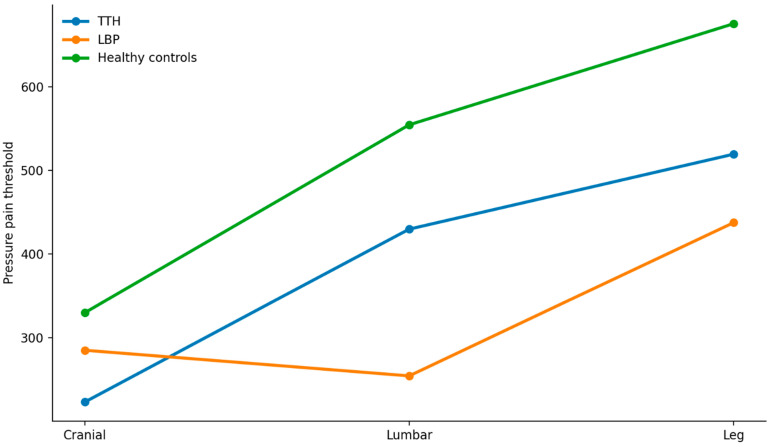
Regional pressure pain threshold profiles expressed in kilopascals (kPa) within study groups.

Within-group analysis showed that the lowest absolute PPT values were consistently observed in the clinically dominant painful region (Figure 2).

In the TTH group, the cranial region had the lowest thresholds, whereas in the LBP group, the lumbar region had the lowest.

Healthy controls demonstrated uniformly higher thresholds across all examined regions.

### 3.2. Secondary Outcomes

Exploratory secondary analyses showed that age and body mass index were inversely associated with PPT values across the examined regions. Age showed inverse correlations with cranial, lumbar, and lower-extremity PPT values (r = −0.152, r = −0.498, and r = −0.405, respectively; all *p* < 0.001). Similarly, BMI was inversely associated with cranial, lumbar, and lower-extremity PPT values (r = −0.160, r = −0.556, and r = −0.441, respectively; all *p* < 0.001). Sex-related differences were modest and inconsistent across regions. Higher pain intensity and longer symptom duration were associated with lower thresholds, particularly within clinically affected regions. In the TTH group, higher monthly headache frequency was associated with lower cranial PPT.

## 4. Discussion

The present study provides a direct quantitative comparison of regional PPT profiles in two clinically distinct chronic pain syndromes examined under identical methodological conditions. Although TTH and LBP differ substantially in anatomical localization and clinical presentation, both groups showed a consistent pattern characterized by the lowest PPT in the primary symptomatic region, with reduced thresholds in anatomically distant areas [13,15,31,32].

The most pronounced regional reduction was observed in the cranial region in patients with TTH and in the lumbar region in patients with LBP, indicating syndrome-specific dominant local mechanical hypersensitivity in the clinically affected segment. This finding is consistent with the concept that chronic pain syndromes are accompanied by increased regional nociceptive sensitivity, most pronounced at the site of persistent symptoms [13,14,15,31,32]. At the same time, both pain groups showed lower PPT outside the primary painful region than healthy controls. Although these extraregional reductions were less pronounced than local changes, their consistent presence across groups indicates that altered mechanical sensitivity was not strictly confined to the clinically dominant area. Reduced PPT values in anatomically distant regions may be consistent with broader alterations in nociceptive processing, including mechanisms associated with central sensitization [3,13,33]. Previous studies in TTH have shown both localized and widespread pressure-pain hypersensitivity, supporting the view that mechanical sensitivity in chronic headache may extend beyond the cranio-cervical region [9,14,34,35]. Similarly, chronic LBP has been associated with altered quantitative sensory testing profiles, reduced pressure pain thresholds, impaired descending pain modulation, and features consistent with centrally mediated pain amplification in at least a subgroup of patients [3,15,33]. However, central sensitization is a complex neurophysiological phenomenon and was not directly assessed in the present study. Therefore, the current findings should be interpreted as evidence of regional and extraregional mechanical hypersensitivity rather than direct proof of central sensitization. Future studies incorporating quantitative sensory testing, conditioned pain modulation, temporal summation, neurophysiological measures, and psychological assessment are needed to clarify the mechanisms underlying these PPT patterns [3,33,36].

In TTH, reduced cranial PPT corresponds well with the established concept of pericranial mechanical hypersensitivity, one of the most reproducible clinical findings in chronic headache disorders. However, the simultaneous reduction observed in the lumbar and lower-extremity regions indicates that altered pain sensitivity may extend beyond the trigeminocervical system [9,14,34,35].

A similar pattern was observed in LBP. As expected, the lumbar region showed the lowest threshold values, but reduced thresholds in the cranial and lower extremities also suggest broader sensitivity changes that cannot be explained solely by local lumbar pathology [3,15,28,31].

The direct comparison of these two pain populations under identical examination conditions strengthens the interpretation by demonstrating that clinically distinct chronic pain syndromes may share a comparable structural pattern of mechanical hypersensitivity: dominant regional hypersensitivity accompanied by measurable reductions in thresholds in distant regions [13,31,32].

Healthy controls maintained the highest pain-pressure thresholds across all examined regions and preserved a stable regional gradient, supporting the interpretation that the observed reductions in patient groups reflect disease-related alterations rather than normal physiological variability. The PPT values observed in healthy controls were within the range reported in previous quantitative sensory studies of asymptomatic adults [37,38].

Secondary analyses showed that age and body mass index were inversely associated with PPT values, although these effects were smaller than the differences observed between diagnostic groups. Sex-related differences were modest and did not materially influence the principal findings.

Importantly, the main group differences in regional PPT values remained significant after adjustment for age, sex, and BMI. This suggests that the observed PPT patterns were not solely explained by baseline demographic or anthropometric differences between groups. Additional patient-only regression models indicated that the diagnosis-related distribution of PPT values was not fully explained by pain intensity or symptom duration. These adjusted analyses strengthen the interpretation that the observed regional and extraregional PPT patterns are associated with the clinical pain syndrome rather than being explained only by demographic or clinical confounding factors.

From a clinical perspective, these results suggest that quantitative pressure algometry may help characterize not only local pain sensitivity but also broader sensitivity patterns in chronic pain populations. From a mechanistic perspective, PPT assessment may serve as a potential quantitative biomarker of mechanical pain sensitivity. In addition to its role as a clinical outcome measure, pressure algometry may contribute to the phenotyping of chronic pain disorders and to monitoring changes in pain sensitivity over time [13,15,16,32].

In this context, quantitative pain phenotyping may be clinically relevant for identifying patients with predominantly localized mechanical hypersensitivity and those with broader extraregional sensitivity patterns. Such differentiation may support more individualized non-operative and rehabilitation-oriented pain management strategies, particularly in chronic musculoskeletal pain conditions where symptoms, disability, and tissue findings do not always correspond linearly. However, the present study was not designed to evaluate treatment response or rehabilitation outcomes, and future longitudinal studies are needed to determine whether PPT-based phenotyping can guide therapeutic stratification [13,21,39].

The present study has several limitations. First, its cross-sectional design does not permit causal inference. Second, although PPT was assessed using a standardized protocol, the assessor was not blinded to group allocation, and formal intra-rater reliability testing was not performed. However, measurements were performed using the same device and standardized anatomical protocol by trained personnel. Third, psychological and behavioral factors that may influence PPT, including anxiety, depression, pain catastrophizing, sleep quality, and fear-avoidance beliefs, were not assessed. In addition, functional disability scales were not included in the correlation analyses, as the present study was focused on regional and extraregional mechanical pain sensitivity rather than functional impairment. Therefore, the potential contribution of these variables to the observed PPT patterns could not be evaluated. Fourth, no formal a priori sample-size calculation was performed, and the sample size was determined pragmatically based on the availability of eligible participants. Finally, PPT remains dependent on individual perceptual responses despite standardized measurement conditions. These limitations should be considered when interpreting the observed patterns of regional and extraregional mechanical hypersensitivity.

Further studies should evaluate whether these PPT profiles may contribute to longitudinal monitoring, phenotyping, and treatment stratification in chronic pain disorders.

## 5. Conclusions

Patients with tension-type headache and low back pain exhibited distinct regional pressure pain threshold profiles, with the lowest threshold values consistently identified in the clinically dominant painful region. These regional and extraregional PPT differences remained significant after adjustment for age, sex, and BMI, and patient-only analyses further supported diagnosis-related differences after accounting for pain intensity and symptom duration.

Both chronic pain groups demonstrated reduced pressure pain thresholds outside the primary symptomatic area, supporting the presence of extraregional mechanical hypersensitivity. These findings may contribute to more structured phenotyping of mechanical pain sensitivity in chronic pain disorders. However, central sensitization should be considered a possible interpretation rather than a directly confirmed mechanism.

## Figures and Tables

**Table 1:** Demographic, clinical, and regional pressure pain threshold values and clinical characteristics of the study groups.

Variable	TTH (*n* = 200)	LBP (*n* = 200)	Healthy Controls (*n* = 200)	*p* Value
Age (years)	44.9 ± 8.4	52.3 ± 6.9	39.5 ± 7.9	<0.001
Female, *n* (%)	132 (66.0)	71 (35.5)	96 (48.0)	<0.001
BMI (kg/m^2^)	26.1 ± 2.3	28.3 ± 1.9	24.4 ± 1.5	<0.001
Pain duration	56.5 ± 26.6	83.8 ± 40.8	—	<0.001
Pain intensity	6.3 ± 1.3	7.0 ± 1.2	—	<0.001
Pain days per month	17.0 ± 6.5	20.2 ± 6.1	—	<0.001
Cranial PPT	223.4 ± 37.6	285.0 ± 35.0	329.8 ± 50.2	<0.001
Lumbar PPT	429.6 ± 52.3	254.5 ± 47.0	554.4 ± 99.6	<0.001
Lower extremity PPT	519.3 ± 69.3	437.4 ± 65.6	675.3 ± 136.5	<0.001

Data are presented as mean ± standard deviation or number (%). Pain intensity was assessed using an 11-point Numerical Rating Scale (NRS), ranging from 0 to 10. PPT = pressure pain threshold; BMI = body mass index; NRS = Numerical Rating Scale.

**Table 2:** Between-group differences in regional pressure pain threshold values.

Region	Pattern of Difference	ANOVA *p* Value	Effect Size (η^2^)
Cranial PPT	TTH < LBP < Healthy controls	<0.001	0.526
Lumbar PPT	LBP < TTH < Healthy controls	<0.001	0.754
Lower extremity PPT	LBP < TTH < Healthy controls	<0.001	0.514

One-way ANOVA was used for overall group comparisons. Post hoc Tukey analysis confirmed significant pairwise differences between all groups in all regions (all *p* < 0.001).

**Table 3:** ANCOVA-adjusted regional pressure pain threshold values across study groups.

Region	TTH Adjusted Mean (95% CI), kPa	LBP Adjusted Mean (95% CI), kPa	Healthy Controls Adjusted Mean (95% CI), kPa	Adjusted *p* Value	Partial η^2^
Cranial PPT	223.1 (217.2–229.0)	285.4 (278.6–292.3)	329.7 (323.1–336.3)	<0.001	0.507
Lumbar PPT	429.7 (419.7–439.7)	254.5 (242.9–266.1)	554.3 (543.1–565.6)	<0.001	0.643
Lower extremity PPT	519.1 (505.5–532.7)	437.2 (421.3–453.0)	675.7 (660.3–691.0)	<0.001	0.397

Values are adjusted means with 95% confidence intervals derived from ANCOVA models adjusted for age, sex, and body mass index. Adjusted *p* values refer to the overall group effect in ANCOVA models. PPT = pressure pain threshold; TTH = tension-type headache; LBP = low back pain; CI = confidence interval.

**Table 4:** Patient-only multivariable regression analysis of diagnosis-related differences in regional pressure pain threshold values.

Outcome	Adjusted Coefficient for LBP vs. TTH, B (kPa)	95% CI	*p* Value
Cranial PPT	+63.9	55.0 to 72.8	<0.001
Lumbar PPT	−173.7	−185.8 to −161.5	<0.001
Lower extremity PPT	−81.4	−97.9 to −64.9	<0.001

Models were adjusted for age, sex, body mass index, pain intensity, and symptom duration. TTH was used as the reference category. The coefficient B represents the adjusted mean difference in PPT values between LBP and TTH. Positive coefficients indicate higher PPT values in LBP compared with TTH, while negative coefficients indicate lower PPT values in LBP compared with TTH. PPT = pressure pain threshold; TTH = tension-type headache; LBP = low back pain; CI = confidence interval.

## Data Availability

The data analyzed in the current study are not publicly available due to privacy and ethical considerations, but may be obtained from the corresponding author upon reasonable request.

## Abbreviations

The following abbreviations are used in this manuscript:

TTH	Tension-type headache
LBP	Low back pain
PPT	Pressure pain threshold
ICHD-3	International Classification of Headache Disorders, 3rd edition
ICD-11	International Classification of Diseases, 11th edition
IASP	International Association for the study of pain
